# Supplementary material for: Complexome profiling of the Chlamydomonas psb28 mutant reveals TEF5 as an early PSII assembly factor
Source: Plant Cell. 2025 Mar 17;37(6):koaf055. doi: 10.1093/plcell/koaf055 (PMC12164590; doi:10.1093/plcell/koaf055)
Supplement: koaf055_Supplementary_Data [file koaf055_supplementary_data.zip › psb28_interactive.zip!/plots/intensity/qualityPlots/peptides/RAB6_Cre01.g047550.t1.2.html]

### Cre01.g047550.t1.2

Trivial name: RAB6  
SeqCoverage [median]: 40.80  
Mass: 23.67 kDa  
  
 

| Sequence | MissedCleavage | Mass [Da] | wtIntensity | muIntensity | Score |
| --- | --- | --- | --- | --- | --- |
| ELNVNFIETSAK | 0 | 1363.70 | 2827.07 | 11461.06 | 161.63 |
| KIAAALPGMESVTQNKQEQMEEVK | 2 | 2658.34 | 0.00 | 446.46 | 69.92 |
| LQLWDTAGQER | 0 | 1315.65 | 7437.34 | 17035.72 | 128.01 |
| LVFLGDQSVGK | 0 | 1161.64 | 0.00 | 3444.79 | 82.29 |
| QSFLNTAR | 0 | 935.48 | 0.00 | 888.10 | 62.55 |
| QVSIEEGDAK | 0 | 1074.52 | 824.13 | 2665.20 | 70.26 |
| SGATTSPLAK | 0 | 931.50 | 576.95 | 1011.92 | 71.50 |
|  |  |  | 11665.5 | 36953.2 |  |
